# A Visit to the American State School for Idiots

**Published:** 1855-04-01

**Authors:** 


### A VISIT TO THE AMERICAN STATE SCHOOL FOR IDIOTS.

(*From the New York Tribune.*)

A FEW years since, the country people in the neighbourhood of the old Bull's Head Tavern, on the Troy road, when they were told it was to be fitted up as a school-house for idiots, shook their heads, and pretty generally agreed that folks who thought natural fools could be taught anything were but little better than natural fools themselves.

The school was opened, however, and has gradually risen in popular favour, until now the old Bull's Head has got too small to accommodate the large number of pupils for whom applications pour in from all parts of the State. The State, to whom no small portion of the credit of making the experiment belongs, is now erecting a large asylum at Syracuse, where the purposes of the institution can be more fully and effectively carried out.

The present building stands a little out of the city limits, a few rods back from the turnpike. It is a plain, unpretending brick house. Inside, it looks very much like any other boarding-school. If you visit it to see its operation, Dr. Wilbur takes you first into a small building, disconnected with the main one. This room you at first, perhaps, conjecture to be a gymnasium, for two ladders run up to the ceiling, while a third is laid across, horizontally, connecting them, and under it is a mattress. There are two square tables, around which are seated perhaps a dozen boys and girls, of six to sixteen years old, apparently playing with the blocks, coloured balls, printed cards, &c., that lie on them. A young man at one, and a young woman at the other, are talking to the children constantly in loud, but cheery, kindly tones, as if stimulating them to go on with their work, or plays, or whatever it is. The pupils do not have the vacant stare, nor the low retreating foreheads, nor exhibit the personal neglect you expected. One or two have unprepossessing faces, and three or four are cross-eyed, but nearly all look intelligent, and all are neat; some quite pretty. You are a novelty to them, and they follow you with gratified eyes, most of them laughing heartily. This is the first indication they give you of being idiots, for were they sane and sensible, they would understand that visitors are not always a matter of rejoicing.

These are the newest comers. Here are one or two, as yet scarcely taught even to use their senses. That girl's eyes wander restlessly over everything in the room, but wave your handkerchief before them, and she will never notice it, or separate it in her vision from the mass of objects that flits before her dull comprehension. Shout at that boy's ear, and he will hardly pay more attention to it than a post. Put an icicle, or red-hot coal in his hand, and he would scream with pain, but he would not know enough to turn his hand over to let it drop. Throw yonder club at him, and he would not lift a finger to save himself, but would laugh insanely as it struck him down, and never knew what hurt him. A desperate task, indeed, to teach these eyes to see, ears to hear, and benumbed brains to think.

The teacher hangs one of the boys on the ladder by the hands. He has but to let go to drop a few inches upon the soft bed underneath. But he has no sense to teach him that. He clings tightly to the round, and perhaps cries at the pain the act gives him, but he does not move. The teacher puts his arm round him and lifts him up, lifts his hand, places it on the next round, and cheers and encourages him in a kind, loud voice. Then the other hand. And so, after repeated lessons, it is at last almost forced upon his sluggish mind, that he can use his hands and feet to reach the floor.

Another, who has been some months at the institution, is called to show his acquirements. His delight runs over out of his eyes, and he breaks into a broad grin at the opportunity. He runs up the ladder, down it, under it, over it, backward, forward, head foremost, feet foremost, and finally throws himself into the teacher's arms, with an exulting burst of irrepressible laughter. He has been taught to do only what can be taught to dogs and cats; but, with less natural intelligence than they, it is a wonder that he can be taught at all.

Another means of rousing and fixing the dormant faculty of attention is by throwing from hand to hand a stick, as boys play "catch." Two who have become expert at this engage in it, with a nervous straining of every muscle, that denotes what a tremendous mental effort the simple act requires, when such intellects are called upon to perform it.

The little circle around the first table are being taught to put, first one white, then one red bead, alternately, upon a string. Then two white, and two red, &c. They do not learn it immediately, nor in an hour, nor a day, perhaps not a week, or a month. But when they *do* learn, they have gained an idea of *number*—the first in all their lives. And when they have learned it—such extravagant joy! When an idea does enter their poor darkened brains, it is like the thought that occurred to the Greek philosopher in his bath, making him leap out, half-dressed, and run through the streets, shouting, "Eureka!"—"I have found it!" Nowhere is there a pupil that is so grateful to you for a new thought as this poor idiot, that has never learned the use of thoughts at all.

Thus the system proceeds. It begins with the simplest of all impressions—the very foundation. Here is a row of circular blocks of different sizes, and there is a row of holes, into which they respectively fit. The idiot is taught to put each in its appropriate place, and thus he makes his first *comparison*, and gets his first idea of *size*. There is a set of red, green, blue, yellow, and white balls and cups. To fit each ball to its proper cup leads him to a comprehension of differences in *colour*. Another set of various shaped blocks teach him form.

When he has advanced thus far he can be taught to recognise a word printed on a card. But he recognises it as you do a face, by its general aspect, not by its component parts. Upside down or right-side up, it is all the same to him. By degrees, he is brought, first to know its meaning, then its separate letters, and then to trace it on the black board. When he can do this, he has reached the threshold on which ordinary children stand when they first go to school. Thenceforth his education is much like theirs, only requiring infinitely more patience and perseverance and gentleness.

Next you pass into the main school-room, where two dozen or more are assembled. Some are sitting at their desks and books. At one end of the room is a class spelling simple words, and at the other, another class, naming places, as they are pointed out on an outline map. The studies are like those of other schools, but not so the pupils. Time after time do the listless ears turn away, and the dull eyes stare in vacant stupidity. But the teacher's whole heart seems to be set on making them comprehend—she rouses, questions, answers, encourages, smiles, nods, and commends, in rapid succession, and with unflagging, gentle patience. "Now, Eddie!" "Quick, Fannie!" "Spell it, dear." "Think, Harry, that's a good boy!" "You can tell *that*, Kitty,"—patting one on the head, smiling encouragement to the other, and clapping her hands to arouse the attention of a third—for all the world as if they were sound asleep, and she was bound to force the information into their drowsy ears and out of their sleepy mouths in order to wake them up. Sound asleep they are, intellectually, and so they would remain, if her look and voice were not every moment reiterating something to arrest and fix their irregular, wandering train of thought.

At last the slow, hesitating answer comes, given with a trembling eagerness of manner, but with the imperfect lisp of early childhood, for few of the idiots, when first brought here, can speak distinctly. Great is Eddie's triumph if the answer happens to be right. Besides the commendations of the teacher, the whole class beams with sympathetic exultation; for in these simple natures there is an implicit, trusting confidence and lack of jealousy that we educated and wise people are strangers to.

Everything in the studies is made as simple as possible. When the name of an object is to be spelled, the object itself is shown, that they may understand the connexion between the word and the thing. Abundance of pictures, maps, globes, and models illustrate the geographical and historical lessons. In short, no pains are spared to strengthen the two faculties, especially weak in idiots—*concentration* and *conception*.

Pass now into the last room. At the black-board a boy of ten is copying an outline drawing with remarkable fidelity. Another will write his own name, and yours, if requested. Another is performing a difficult sum in long division. Here is a girl of fourteen who cannot speak the simplest word without more exertion that it would cost you to halloo across the street, yet she will name the different countries as you point them out on an outline globe, describe their inhabitants, productions, and physical condition. There is a boy who, besides his idiocy, was pronounced deaf and dumb from his cradle, and came here from the Asylum for Mutes; yet, in less than three years, he has learned the elements of English grammar, and will parse you a sentence and give the syntax. And here is another little fellow with a paralysed arm, who can set down and work out an algebraic formula better than most boys who possess all their faculties. When the idiot can master grammar and mathematics, it is clear that he is an idiot no longer. He can carry out a train of reasoning and reflection, and Plato and Newton had no different process whereby to attain the greatest philosophic truths.

It seems strange, and yet it strikes you that somehow these advanced pupils have a more staid and sober look than those whom you saw at first. But so it is. As they exchange a mere animal nature for a human one, they gradually lose that perpetual manifestation of glee so characteristic of idiotcy. It is not that they have made intellectual progress at the expense of physical, for they are plump and rosy. It is not that their development, opening as it does, sources of enduring and deep happiness, has made them grave. But it is beccause a wise Providence partially compensates the poor unfortunate who lacks everything else, by the pleasing, ludicrous images that occupy his vision, and dance in perpetual succession before his bewildered brain.

Of course, while the education of the intellect goes on, that of the moral sense is not neglected. Moral duties are inculcated at each step, and such spiritual truths taught as can be made comprehensible.

The physical teaching and exercise are not the least important part of the school. From being helpless, brutish almost in habits, they are taught to stand, to sit, to walk, to use their hands, to feed themselves, to take care of their persons and clothes, and to conduct themselves like other reasoning beings. One exercise in which the boys take an especial delight is the military manual, which they go through with at the word of command, drawn up in a line, with mimic guns. Sometimes the company is put under command of one of their own number. In the summer they work in the garden, &c.

At meal-time they enter the dining-room quietly and in order, and find and take their own seats. If you look in upon the row, with their neat aprons, clean faces, and smoothly brushed hair, sitting patiently and decorously until they are helped to the dishes before them, you would hardly believe that they belong to the wild, uncontrollable class of beings that are commonly known as idiots.

That the discipline is firm and strict, you cannot but believe, on seeing these effects of it, and on watching the ready obedience yielded to the teachers. Yet that it is marked by parental gentleness and kindness cannot be doubted, when you see with what eagerness they comply with their teachers' wishes, with what satisfaction they receive their approval, how they turn to them in every difficulty or fear, and what affectionate regard they exhibit for each and all of them. One of the teachers told us she found them (saving lack of comprehension) easier to manage than other children of their age. Certain it is, that few schoolmasters can enter their recitation-rooms, assured of so joyful and affectionate a reception as that which greets the entrance of Dr. Wilbur. Not only the State, but the world, owes him a debt of gratitude for his successful experiment, which we trust is yet to be the means of lifting up into the scale of humanity many a poor being hitherto left in mental darkness and bodily misery.

